# Lingual Abscess in an Adult Patient With Pierre Robin Sequence: A Case Report

**DOI:** 10.7759/cureus.65460

**Published:** 2024-07-26

**Authors:** Hiram H Plata-Huerta, Astrid E Rosero-Castillo, Jose Luis Trevino Gonzalez

**Affiliations:** 1 Otolaryngology - Head and Neck Surgery, Hospital Universitario Dr. José Eleuterio González, Universidad Autonoma de Nuevo Leon, Monterrey, MEX

**Keywords:** pierre robin sequence, oral and maxillofacial pathology, syndromic diseases, ent surgeon, ent infections, head and neck imaging, lingual abscess

## Abstract

A lingual abscess is a rare but serious infection within the tongue parenchyma, posing significant risks due to potential airway obstruction. Despite advancements in oral hygiene and antibiotics, timely diagnosis and treatment are critical to prevent severe complications. In this case, we report a 29-year-old male with Pierre Robin sequence (PRS) who presented with a four-day history of severe tongue pain, swelling, decreased appetite, and fever, without any reported trauma. Examination revealed left-sided tongue swelling, poor oral hygiene, and notable Mallampati III classification. A neck CT scan confirmed an abscess in the left hemitongue involving the intrinsic and mylohyoid muscles, measuring 26.5 x 30 x 30.5 mm with a volume of approximately 8 cc. Prompt intravenous antibiotic treatment was initiated, leading to spontaneous abscess drainage and significant clinical improvement. The patient was discharged after five days of intravenous antibiotics and continued oral antibiotics. At one-week follow-up, he was asymptomatic and fully recovered. This case underscores the importance of recognizing the potentially life-threatening nature of lingual abscesses, particularly in syndromic patients like those with PRS, who may experience quicker airway obstruction due to craniofacial abnormalities, such as micrognathia and glossoptosis. Given the rarity of such conditions, awareness and readiness to address these emergencies are essential for ensuring patient safety and positive outcomes.

## Introduction

A glossal abscess is a relatively uncommon but serious infection within the tongue parenchyma, potentially leading to severe or life-threatening consequences [1,2]. In past decades, these abscesses were more common due to poor oral and dental hygiene standards [3]. Their formation results from a weakened immune system and breaches in the mucosal barrier of the tongue. Contributing local factors include poor oral hygiene, dental caries, recent dental surgery, smoking, alcohol consumption, and foreign body trauma. In addition, infections in the tonsils, nasopharynx, and floor of the mouth are frequently observed [1,4,5]. Cultures often grow *Streptococcus* species, *Staphylococcus* species, and diphtheroids; moreover, blood cultures of lingual cellulitis can also yield *Klebsiella* and *Streptococcus* species [6].

Glossal abscesses are categorized based on their location as anterior (anterior two-thirds of the tongue) or posterior (posterior third of the tongue). Anterior abscesses typically originate from infections related to dental caries, periodontal disease, or trauma, while posterior abscesses originate from infections of lingual tonsils, remnants of thyroglossal duct cysts, or molars [7-9]. They typically present with marked pain, impaired speech, sialorrhea, and glossitis, which can manifest as generalized or unilateral swelling. Examination may reveal a range of findings, from soft, fluctuant to indurated homogeneous masses with diffuse erythema or exudates, as well as variable pain presentations. Differential diagnoses include metastatic tongue lesions with similar presentations [7-8]. The time from symptom onset to seeking medical attention has been reported as up to seven days, although it can be several weeks [6,10-13].

There is a scarcity of literature regarding glossal abscesses. For example, Rampi et al. reported 47 published cases since 1999, while Antoniades et al. reviewed approximately 50 cases over the last 30 years. Prior to these, knowledge was mainly derived from short case reports, many dating back to the pre-antibiotic era [14]. Despite advancements in diagnostic technology, limited data are available on whether outcomes for this disease have improved due to the rarity of reported cases. Pierre Robin sequence (PRS) refers to the association of micrognathia, glossoptosis, a spectrum of cleft palate presentation, and airway obstruction [15]. Herein, we report the case of a 29-year-old male with PRS, characterized by the common features of glossoptosis and micrognathia, who was diagnosed with and successfully treated for an anterior lingual abscess.

## Case presentation

**Figure 1:**
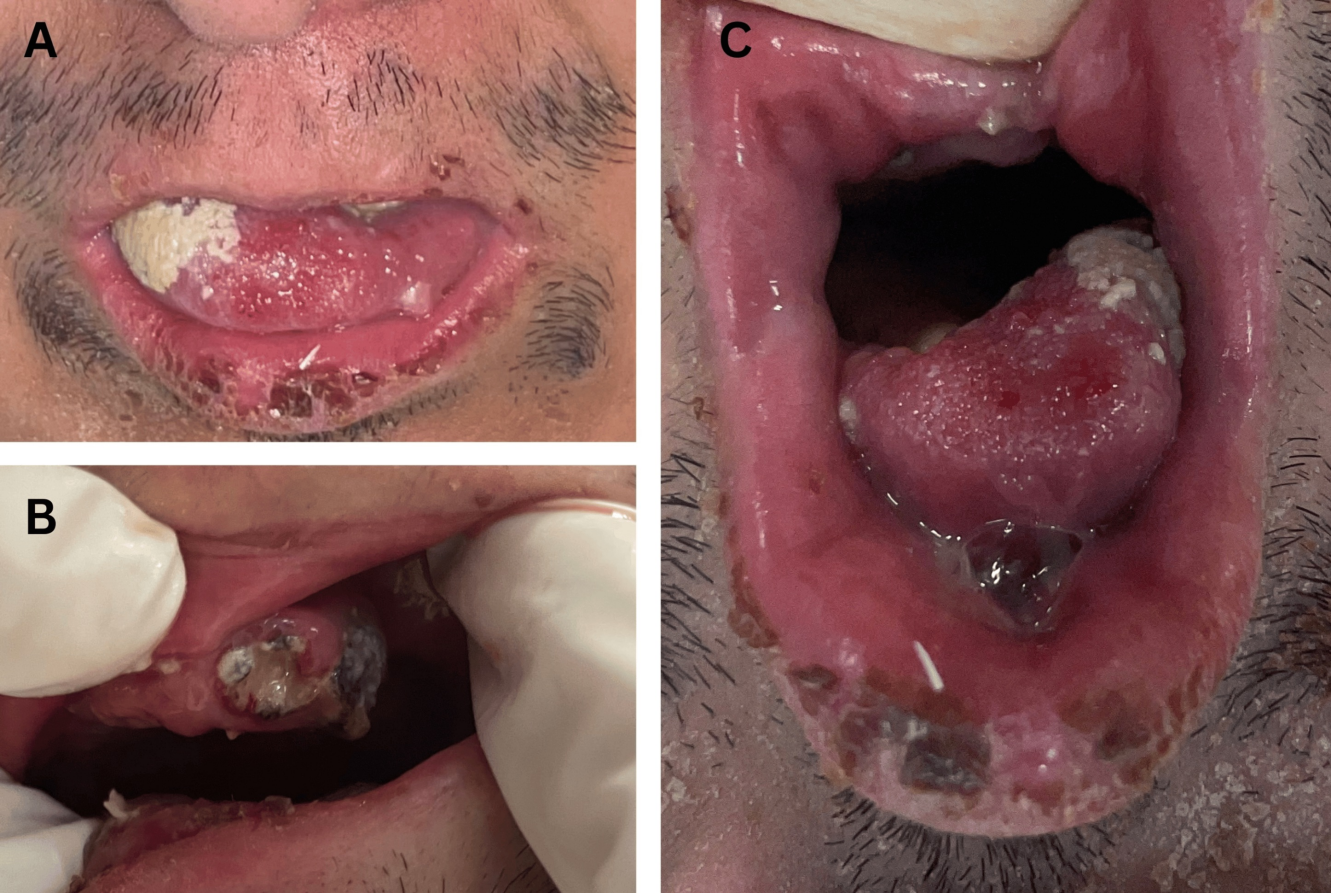
Tongue presentations and oral hygiene on initial evaluation of the patient. (A) Tongue swelling with micrognathia. (B) Poor oral hygiene, partial edentulism, and stage-5 caries. (C) Tongue swelling and edema on the left side.

A 29-year-old male with a diagnosis of PRS was admitted to the emergency room with a four-day history of severe tongue pain, swelling, edema, decreased appetite, and fever without any reported tongue trauma. Physical examination revealed tongue swelling on the left side, a painful exploration with soft edema of the oral floor (Figure 1A, 1C), micrognathia, precarious oral health, partial edentulism, and stage five dental caries (Figure 1). No fever, localized hyperthermia, trismus, or respiratory distress were noted. The laboratory and blood gas analyses, as described in the literature, revealed elevated levels of acute phase reactants, including leukocyte count and CRP level (Table 1) [7].

**Table 1:** Laboratory and blood gas findings CRP: C- reactive protein, ESR: erythrocyte sedimentation rate

Parameter	Values	Range (unit)
Laboratory results		
Hemoglobin	15.6	12.2-18.1 (G/dL)
Leukocytes	13.2	4.00-11.00 (K/UL)
Neutrophils	10.1	2.00-6.90 (K/UL)
Lymphocytes	2.08	0.60-3.40 (K/UL)
Platalets	283	142.00-424.00 (K/UL)
CRP*	10.3	0-1.00 (mg/dL)
ESR*	38	0-9 (mm/hr)
HIV-1/2	NEGATIVE	
Blood gas analysis		
pH	7.45	7.32-7.43
pCO2	34	40-45 (mmHg)
HCO3	23.6	34.0-30.0 (mmol/L)

**Figure 2:**
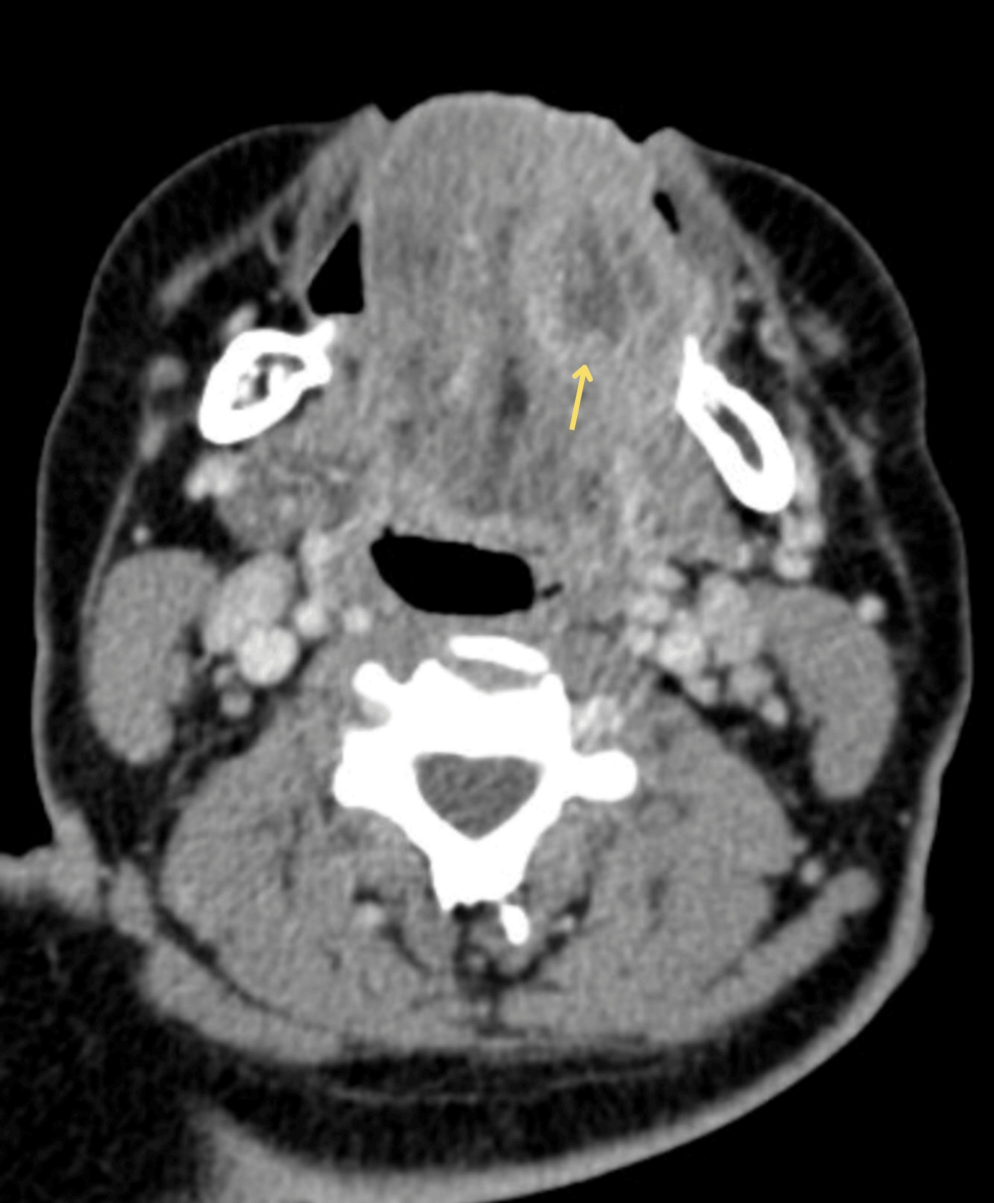
Contrast CT neck scan Abscess in the left hemitongue involving the intrinsic muscles (yellow arrow) shows collection with periferial enhancement.

**Figure 3:**
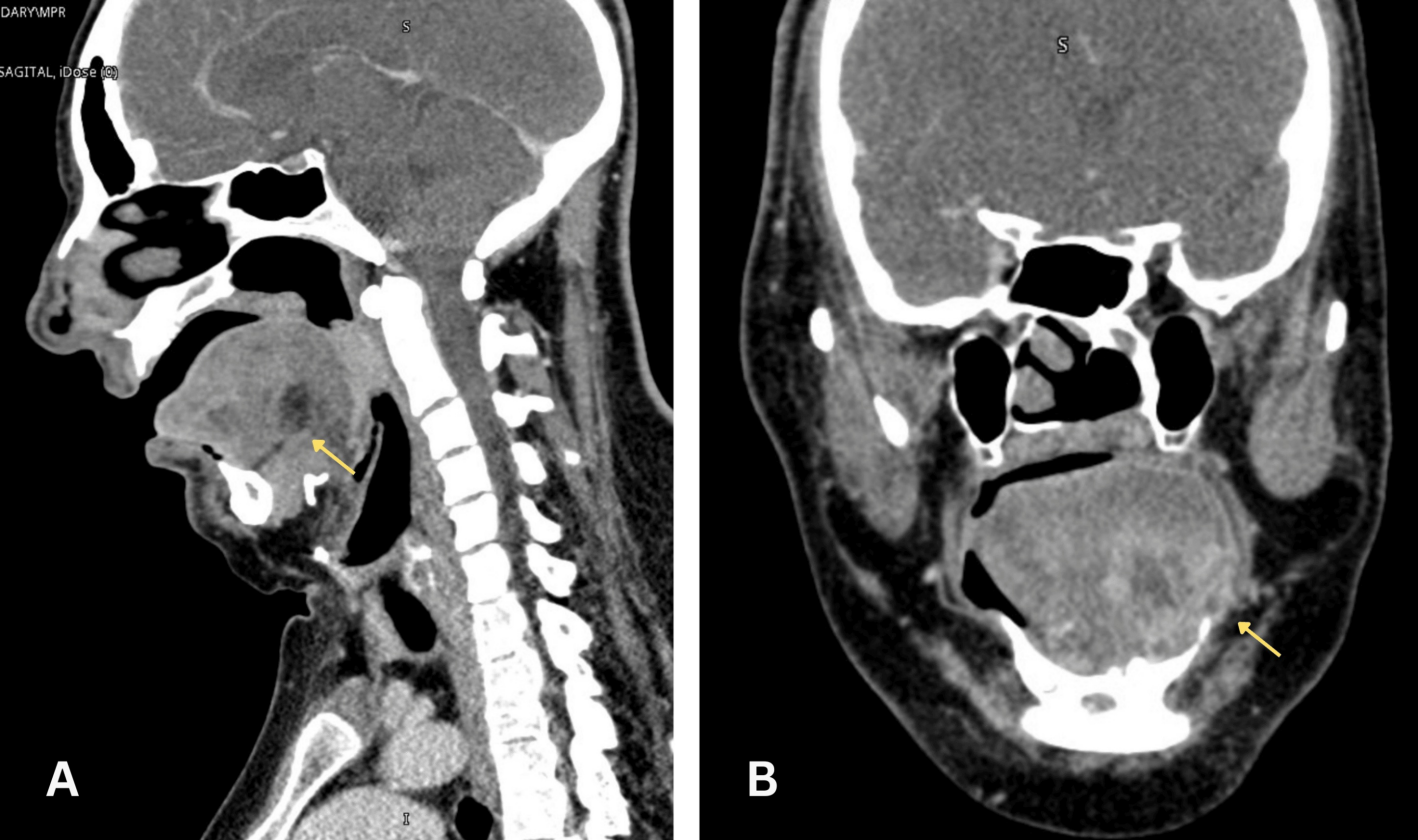
Contrast CT neck scan. Collection in the left hemitongue involving the intrinsic muscles. (A) Sagittal and (B) coronal window (yellow arrow) show collection with periferial enhancement.

A neck contrast CT scan was promptly performed, which identified a collection in the left hemithongue involving the intrinsic muscles of the tongue (vertical, transverse, and inferior longitudinal), as well as the mylohyoid muscle on the same side, without compromising the submandibular space or glandular structures, consistent with the clinical findings previously described. The collection measured 26.5 x 30 x 30.5 mm, with an approximate volume of 8 cc. Multiple cervical lymph nodes appeared reactive in IB, IIA, and III levels bilaterally (Figure 2). The cervicodorsal spine showed altered fusion (butterfly vertebrae), and prominent tonsils reduced the airway space in the oropharynx. In addition, the ear examination revealed stenosis of the right vestibular aqueduct (Figure 3).

We promptly initiated intravenous ceftriaxone 1 gr bid and metronidazole 500 mg tid and analgesics. The abscess spontaneously drained through the ipsilateral edge of the tongue, although the fluid was not collected. The patient showed clinical improvement and was discharged after five days of intravenous antibiotics, with a prescription for oral antibiotics based on amoxicillin/clavulanate 875/125 mg bid and metronidazole 500mg tid, with the intention to continue a board-spectrum approach. At a follow-up consultation one week later, he was asymptomatic and fully recovered.

## Discussion

A lingual abscess is an infectious process within the tongue parenchyma. It is a rare condition since the advent of antibiotics, with mortality of up to 3%. Due to the risk of airway obstruction, it should be recognized and treated as an airway emergency as soon as possible [5]. It usually presents with painful swelling that causes protrusion of the tongue, dysphagia, odynophagia, slurred speech, or respiratory distress where mucosal trauma is its main etiology. In this case, our patient had no history of mucosal trauma; therefore, poor oral hygiene may have been the predisposing factor [10]. Healthcare providers must recognize the potentially life-threatening nature of lingual abscesses, as they can be a serious medical condition [1,16]. The airway is typically secure; however, anticipating the possibility of airway deterioration is essential. The timing of symptom onset can vary, with some individuals experiencing symptoms within a few days and others waiting weeks. A delay in diagnosis can lead to complete airway obstruction, hindering prompt intubation by medical providers [4,16].

The most frequent treatment approach is initially empirical broad-spectrum antibiotics to cover the most common *Streptococcus*, *Staphylococcus* species, and diphtheroids with a complement of surgical drainage [6,16]. Our patient exhibited a site of spontaneous drainage. It was managed with continuous and rigorous monitoring for airway obstruction, and a broad empirical antibiotic therapy was promptly initiated. Syndromic patients pose a particular challenge due to the unknown natural history, pathology, and comorbidities they may present. The presentation of this PRS case, characterized by micrognathia, glossoptosis, and the presence of an oral abscess, is an element that necessitates prompt management of airway obstruction. Surgical drainage in these cases is urgent; in this instance, the presentation of sudden drainage was beneficial for medical management [17,18]. Hence, we present this rare case in a syndromic patient to emphasize the importance of recognizing and understanding uncommon abscesses in specific patient populations and to underscore the critical necessity of timely intervention.

## Conclusions

This case described the presentation of a non-traumatic anterior lingual abscess in a male with PRS. The presentation featured marked, soft, and fluctuant unilateral swelling. The significant potential for airway obstruction is a crucial aspect of this case. Rapid healing achieved with medical treatment, without the need for drainage, underscores the importance of timely diagnosis and intervention. We strongly advise physicians to remain vigilant and recognize the potentially life-threatening nature of lingual abscesses. Age at presentation, specific craniofacial malformations, and the stage of comorbidities were crucial factors influencing the positive outcome in this case. It is important to consider that most emergency physicians and head and neck surgeons may never encounter these conditions in their practice.
